# Cell-intrinsic and tumor microenvironmental determinants of platinum resistance in epithelial ovarian cancer

**DOI:** 10.1007/s12672-026-04702-0

**Published:** 2026-02-27

**Authors:** Wutao Chen, Weiwei Shi, Hoi Lam Chan, You Wang, Wen Di

**Affiliations:** 1 Department of Obstetrics and Gynecology, Renji Hospital, School of Medicine, Shanghai Jiao Tong University, 200127 Shanghai, China; 2 Shanghai Key Laboratory of Gynecologic Oncology, Renji Hospital, School of Medicine, Shanghai Jiao Tong University, 200127 Shanghai, China; 3 State Key Laboratory of Systems Medicine for Cancer, Renji Hospital, School of Medicine, Shanghai Jiao Tong University, 200127 Shanghai, China

**Keywords:** Ovarian cancer, Chemotherapy, Platinum, Drug resistance, Tumor microenvironment

## Abstract

Epithelial ovarian cancer (EOC) remains the most lethal gynecologic malignancy, primarily due to late-stage diagnosis, high relapse rates, and the inevitable emergence of platinum resistance. Although platinum-based chemotherapy produces high initial response rates, over 70% of patients eventually relapse with tumors that are refractory to treatment. Platinum resistance develops through complex and dynamic mechanisms involving both tumor-intrinsic and tumor-extrinsic factors. Tumor-intrinsic pathways include enhanced DNA repair, apoptosis evasion, metabolic reprogramming, and transcriptional or epigenetic plasticity, while tumor microenvironmental influences such as hypoxia, immune suppression, and stromal remodeling further sustain resistant phenotypes. This review highlights recent methodological and conceptual advances that have reshaped the understanding of platinum resistance in EOC. We summarize state-of-the-art experimental platforms ranging from two- and three-dimensional culture systems to lineage-tracing technologies, single-cell sequencing, and integrated multi-omic strategies. Together, these approaches enable dynamic mapping of clonal evolution and adaptive responses under therapeutic pressure. We also discuss emerging therapeutic strategies aimed at overcoming resistance, including inhibitors of DNA damage response, antibody–drug conjugates, anti-angiogenic agents, immunotherapies, and targeted drug delivery systems. Recent clinical advances illustrate the translational potential of mechanism-based interventions. Despite these promising developments, durable clinical responses remain limited, emphasizing the need for biomarker-guided combination therapies. An integrated, multi-omics–driven, and patient-tailored framework will be critical for re-sensitizing resistant tumors and improving long-term outcomes in ovarian cancer.

## Introduction

Epithelial ovarian cancer (EOC) presents significant challenges due to its typically late diagnosis, high recurrence rate, and poor long-term survival outcomes [1]. The standard treatment approach combines cytoreductive surgery with platinum-based chemotherapy. In advanced cases, neoadjuvant chemotherapy followed by interval cytoreductive surgery may offer comparable survival benefits [2]. While most patients initially respond well to first-line chemotherapy, over 70% experience recurrence and develop resistance to platinum-based treatments, necessitating alternative therapies, posing a significant clinical challenge and contributing to poor long-term survival outcomes [3].

The persistence of platinum resistance reflects the complexity of EOC biology. A key challenge in understanding chemoresistance is determining how a small subset of tumor cells survives treatment while the majority succumb. Platinum resistance is broadly governed by two intertwined domains: cell-intrinsic mechanisms, such as enhanced DNA repair and altered drug metabolism, and tumor microenvironment (TME) influences, including stromal remodeling, immune suppression, and metabolic crosstalk. Despite intensive investigation, clinically actionable strategies to reverse resistance remain limited, underscoring the need for deeper insight into how these intrinsic and extrinsic factors co-evolve to support drug tolerance.

Platinum resistance in ovarian cancer can be divided into primary and acquired resistance in biological settings. Primary resistance is thought to stem from pre-existing heterogeneity within a tumor: rare subpopulations or stress-tolerant cells that harbor survival advantages can survive first-line therapy. In contrast, acquired resistance evolves under drug pressure, through transcriptional reprogramming, epigenetic shifts, or selective adaptation of surviving cells [4, 5]. Tumors with dominant intrinsic resistance may not benefit from continued platinum rechallenge, whereas in those with acquired resistance, strategies targeting adaptive pathways may resensitize cells [6–8].

Recent advances in single-cell RNA sequencing (scRNA-seq), spatial transcriptomics, spatial proteomics, and patient-derived models have begun to reshape our understanding of chemoresistance—not as a static trait, but as a dynamic and heterogeneous process shaped by both tumor cell plasticity and microenvironmental cues. Moreover, novel therapeutic strategies, including nanotechnology-based delivery systems, epigenetic modulators, and tumor-targeted immune therapies, are being actively explored to circumvent platinum resistance.

This review aims to provide a comprehensive overview of cell-intrinsic mechanisms being explored to combat chemoresistance, as well as the components of the TME that may contribute to this phenomenon. We seek to highlight current challenges, identify knowledge gaps, and propose future directions for improving outcomes in platinum-resistant ovarian cancer.

### Two models of platinum resistance: clinical and biological

In clinical settings, platinum resistance has traditionally been categorized as either platinum-sensitive disease (recurrence ≥ 6 months after initial platinum-based treatment) or platinum-resistant disease (recurrence within 6 months after initial platinum-based treatment) [9, 10]. While clinically useful, this classification has been increasingly questioned due to its reliance on arbitrary time cutoffs and its inability to reflect the underlying biological diversity of resistance mechanisms [11–13].

More detailed terms are used in recent papers. The platinum-free interval (PFI) is the time from the last dose of the most recent platinum-based regimen to disease progression, whereas the primary platinum-free interval (PPFI) is measured from the end of first-line platinum therapy [14]. Resistance is also split into primary platinum resistance (PRR)—progression < 6 months after first-line platinum—and secondary platinum resistance (SRR)—progression ≥ 6 months after first-line platinum but < 6 months after the subsequent platinum-based therapy [9, 15, 16].

Given that nearly all patients with advanced EOC ultimately develop resistance to platinum, some investigators propose a more biologically grounded framework—distinguishing between pre-existing (primary) resistance and adaptive (acquired) resistance. This paradigm shift is supported by recent evidence suggesting that resistance is not a binary trait, but a continuum shaped by tumor heterogeneity, clonal dynamics, and microenvironmental pressures [17, 18].

#### Primary resistance

Support for primary resistance stems from the observation that EOC tumors are often polyclonal at diagnosis. Lineage tracing and single-cell studies have revealed the presence of rare, stress-tolerant cell states within treatment-naïve tumors that preferentially survive chemotherapy [19–21]. Tools such as ReSisTrace [12] and Watermelon lineage barcoding [22] have demonstrated that resistant clones are not randomly generated during treatment, but rather are selectively enriched from pre-existing populations following platinum exposure. These findings suggest that primary resistance may arise from inherent cellular programs—such as elevated DNA repair capacity, stem-like phenotypes, or epigenetic plasticity—present before treatment initiation.

#### Acquired resistance

Conversely, acquired resistance reflects an evolutionary response to therapy-induced stress. Tumor cells may undergo transcriptional reprogramming, chromatin remodeling, or phenotypic switching in response to chemotherapy. Notably, lineage plasticity, wherein tumor cells transdifferentiate into alternative histological subtypes, has been observed in lung adenocarcinoma, prostate cancer, and bladder cancer as a resistance mechanism to targeted therapies [23–25]. Although this phenomenon is less characterized in ovarian cancer, accumulating evidence indicates that acquired platinum resistance in ovarian cancer is driven by widespread reprogramming of transcriptional and epigenetic programs [21, 26–28]. Across ovarian cancer cell lines and patient samples, chemotherapy has been shown to activate inflammatory and stress-response pathways [29], alter DNA methylation [30–32], remodel chromatin accessibility [33], and shift metabolic and signaling networks in surviving cells [34]. These transcriptomic and epigenomic adaptations suggested that platinum resistance frequently reflects adaptive rewiring of cellular programs. However, lineage plasticity does not account for all resistance patterns. For instance, BRCA1/2 mutations are well known to confer increased platinum sensitivity [35], whereas CCNE1 amplification is frequently associated with reduced platinum responsiveness [36].

**Fig. 1:**
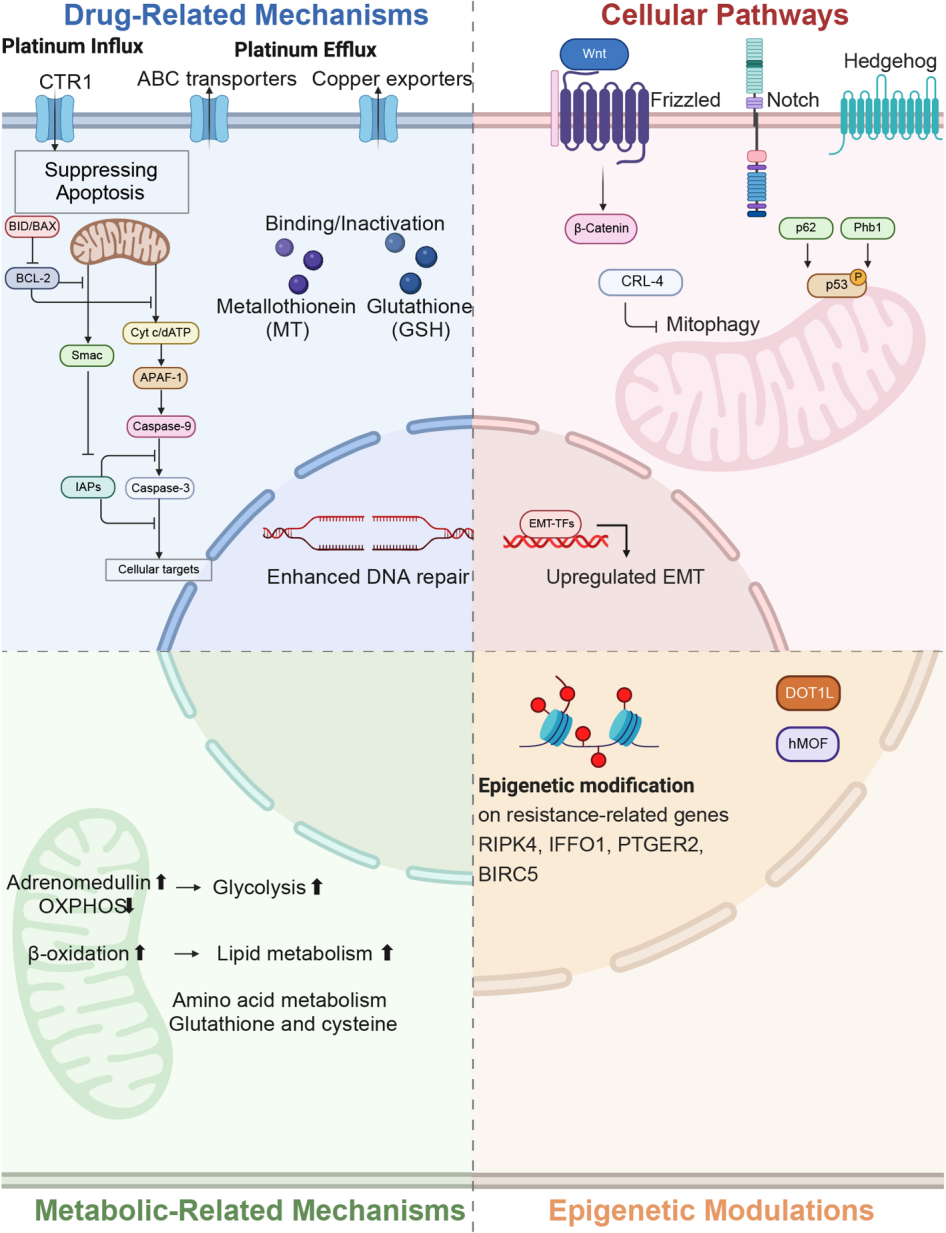
Cell-Intrinsic Mechanisms of Platinum Resistance. Graphical overview of significant mechanisms contributing to platinum resistance in cancer cells. Key drug-related mechanisms include increased efflux via ABC transporters, reduced influx through CTR1 downregulation, and inactivation by GSH, MT, and GST. Enhanced DNA repair pathways also play a crucial role. Suppression of the pro-apoptotic proteins would promote cell survival while suppression of anti-apoptotic proteins would promote cell death. Cellular pathways, particularly Wnt, Notch, and Hedgehog, regulate cancer stem cell properties and EMT. Mitochondrial functions in mitophagy (CRL4) and apoptosis regulation (p53, p62, Phb1) are implicated in resistance. Metabolic reprogramming, including shifts in glucose, lipid, and amino acid metabolism, alters drug response. Epigenetic modifications, such as DNA methylation and histone alterations (regulated by DOT1L and hMOF), contribute to the development of resistance. [created with BioRender.com (https://biorender.com/)]

Despite the apparent dichotomy, these models are not mutually exclusive. Many resistant tumors likely harbor a mixed composition of intrinsically resistant clones and cells that acquire resistance over time. Elucidating the interplay between these mechanisms is essential for developing personalized interventions that prevent or delay resistance emergence (Fig. 1).

### Mechanism of platinum-based drugs

Platinum-based chemotherapy agents, such as cisplatin and carboplatin, are cornerstone treatments in ovarian cancer and many other malignancies. Their cytotoxic effects are primarily attributed to their ability to form intra-strand and inter-strand crosslinks with DNA, particularly at the N7 position of guanine. These crosslinks obstruct DNA replication and transcription, ultimately triggering cell cycle arrest and leading to apoptosis through the activation of DNA damage response pathways [37]. Recent research has also uncovered additional modes of action, including disruption of RNA transcription, interference with oncogenic proteins, alteration of cellular metabolism, and induction of cytoplasmic stress responses including oxidative stress, calcium imbalance, and breakdown of protein quality control [38–42].

### Cell-intrinsic mechanisms of platinum resistance

#### Drug-related mechanisms

Resistance to platinum-based chemotherapy in ovarian cancer often originates from adaptive alterations in the way cancer cells process and respond to the drug. These adaptations can occur at multiple levels, including impaired drug accumulation, enhanced DNA repair capacity, and evasion of apoptosis. Together, these mechanisms enable tumor cells to tolerate platinum-induced cytotoxicity and survive treatment.

- Impaired drug accumulation and detoxification

The intracellular concentration of platinum agents is a critical determinant of therapeutic efficacy. This balance is regulated by membrane transporters that mediate both drug influx and efflux. CTR1 plays a pivotal role in mediating the uptake of cisplatin and carboplatin. Its downregulation is commonly observed in resistant ovarian cancer cells and correlates with poor drug accumulation and treatment failure [43, 44]. Conversely, drug efflux is primarily mediated by ATP-binding cassette (ABC) transporters, including ABCB1 and ABCC2, which actively export platinum compounds out of the cell. Overexpression of these transporters has been linked to platinum resistance in both preclinical and clinical studies [45, 46].

Once inside the cell, platinum compounds can be rendered inactive through binding to thiol-containing molecules such as GSH and MT. These proteins act as scavengers, sequestering platinum compounds and preventing their interaction with DNA [5]. Recent findings highlight the role of GSTs—enzymes that catalyze GSH conjugation—as key mediators of this detoxification process. Overexpression of GSTT1, in particular, has been associated with platinum resistance and poorer outcomes in ovarian cancer [47]. MT expression levels in tumor samples have also emerged as potential biomarkers for predicting platinum response [48].

b) Enhanced DNA Damage Repair

Many ovarian cancer cells evade platinum-induced apoptosis by activating DNA damage response (DDR) [49]. Among DDR mechanisms, homologous recombination repair (HRR) is clinically the most critical in ovarian cancer. Restoration of HRR—for instance via BRCA1/2 reversion mutations, promoter demethylation, or upregulation of core effectors like RAD51—enables tumor cells to repair platinum-induced DNA lesions and evade apoptosis [19, 50]. In clinical cohorts, patients harboring germline or somatic BRCA1/2 mutations show significantly better responses to platinum chemotherapy, but secondary reversion events that restore HR are a well-established mechanism of resistance [51–53].

Other DDR pathways, including nucleotide excision repair (NER) and mismatch repair (MMR), also influence platinum sensitivity—but their clinical impact appears modest. Loss of MMR or NER proficiency sensitizes tumors to platinum, whereas restoration of these pathways facilitates resistance. Notably, NER defects are associated with improved overall and progression-free survival in EOC patients [54]. However, the prognostic value of ERCC1, a core NER component, remains controversial, as studies have yielded inconsistent results regarding its association with platinum sensitivity [54–57].

c) Apoptosis Evasion

When unresolved DNA damage accumulates after platinum exposure, tumor cell death classically proceeds via the intrinsic apoptotic cascade. However, many ovarian cancer cells circumvent apoptosis by shifting the balance of BCL2 family proteins [58, 59]. Among anti-apoptotic genes, BCL-XL is more frequently deregulated in ovarian cancer than BCL2, making it a dominant survival factor in many settings [60–64]. Overexpression of BCL-XL, or loss of pro-apoptotic effectors like BAX, suppresses mitochondrial permeabilization and downstream caspase activation, contributing to platinum resistance [61, 65–67]. Another example is the stabilization of MCL1. Deubiquitinases such as JOSD1 and DUB3 prevent MCL1 degradation, sustaining its expression and blocking mitochondrial outer membrane permeabilization. In this way, MCL1 overexpression further suppresses apoptosis and enhances resistance to platinum-induced cytotoxicity [68, 69].

#### Cellular pathways

Beyond drug accumulation and DNA repair, platinum resistance in ovarian cancer is profoundly shaped by intracellular signaling pathways and cellular phenotypic plasticity. These processes govern how tumor cells adapt to stress, evade apoptosis, and maintain survival under chemotherapeutic pressure. Notably, these pathways are not isolated—they often interact and reinforce one another, creating a multidimensional resistance network.

- Cancer Stemness and Self-Renewal Pathways

Cancer stem cells (CSCs) represent a subpopulation of tumor cells with self-renewal capacity and the ability to drive recurrence after treatment. Their quiescent state and high DNA repair proficiency make them inherently resistant to cytotoxic agents, including platinum compounds [70]. In ovarian cancer, both the ovarian surface epithelium (OSE) and fallopian tube epithelium (FTE)—the presumed origins of high-grade serous ovarian cancer—express stemness-associated genes such as NANOG, SOX2, and ALDH1A1 [71, 72]. Clinically, elevated CSC marker expression is linked to poor overall survival (OS), progression-free survival (PFS), and disease-free survival (DFS) [73, 74].

Several developmental signaling pathways regulate CSC maintenance: Wnt signaling, particularly via FZD receptors, has been implicated in platinum resistance. Overexpression of FZD3, FZD5, and FZD7 supports stem-like states and promotes drug tolerance [75–78]. Notch signaling, driven by ligands such as Jagged2 and receptors like Notch1/2, fosters CSC enrichment and correlates with resistance and metastasis. CD109 has also been reported to enhance Notch-mediated stemness [79]. Hedgehog signaling, particularly the Gli-NANOG axis, maintains pluripotency and reinforces drug resistance by preserving stemness and inhibiting differentiation [80–83].

Emerging evidence suggests that CSCs may not be a fixed population but rather arise through dynamic interconversion between differentiated and stem-like states—a process influenced by the tumor microenvironment and therapeutic stress [84]. Understanding these transitions is critical for developing therapies that prevent or reverse stemness-driven resistance.

b) Mitochondrial Adaptions

Mitochondria act as both metabolic regulators and stress sensors, mediating apoptosis, redox homeostasis, and mitophagy. These functions become pivotal under chemotherapy-induced stress, especially during platinum exposure [85, 86]. Platinum resistance in ovarian cancer has been linked to mitochondrial adaptation and remodeling, including: Mitophagy induction: Degradation of damaged mitochondria through autophagy is a survival strategy. Knockdown of *CRL4* enhances mitophagy in resistant cells, promoting survival under cisplatin treatment [87]. Suppression of mitochondrial apoptosis: Platinum-induced apoptosis is largely mediated by p53 localization to mitochondria, which facilitates cytochrome c release. Proteins like p62 and Phb1 regulate p53’s mitochondrial function, modulating the apoptotic threshold [88, 89]. These mitochondrial mechanisms illustrate how metabolic and apoptotic regulation converge to support chemoresistance in ovarian cancer.

c) Epithelial-Mesenchymal Transition (EMT)

EMT is a reversible biological program through which epithelial cells acquire mesenchymal traits, including motility, invasiveness, and survival advantage. In ovarian cancer, EMT not only facilitates metastasis but also contributes to chemoresistance [90–93]. Platinum exposure can induce EMT-like changes in ovarian cancer cells, leading to enhanced tolerance to DNA damage and reduced drug sensitivity [94, 95]. Recent transcriptomic profiling has identified EMT-promoting genes such as: *MAP7*, which modulates microtubule dynamics and promotes invasion, *ITGA6*, involved in adhesion and stemness, *TMEM200A* and *PRKAR1B*, which regulate intracellular signaling cascades implicated in resistance [94, 96, 97]. Although these markers are associated with platinum resistance, their functional roles in EMT-induced drug tolerance remain incompletely understood. Further research is needed to delineate how EMT interacts with other pathways and whether targeting EMT regulators can sensitize tumors to chemotherapy.

#### Metabolic-related mechanisms

Platinum-resistant ovarian cancer cells often reprogram their metabolism to survive therapeutic stress [98, 99]. These adaptations enable cells to generate energy, scavenge oxidative stress, and maintain biosynthetic capacity in hostile conditions. Recent studies highlight alterations in glucose metabolism, lipid utilization, and amino acid pathways as key features of platinum resistance.

- Glucose Metabolism

Rewiring of glycolytic pathways is a hallmark of chemoresistant cells. Tumors with low oxidative phosphorylation (OXPHOS) tend to rely heavily on aerobic glycolysis, which supports resistance through enhanced energy flux and reduced oxidative stress. In contrast, high-OXPHOS tumors accumulate reactive oxygen species and are more susceptible to platinum-induced damage [100]. One notable regulator is adrenomedullin (ADM), a hypoxia-inducible gene that enhances glycolysis and reduces drug sensitivity. ADM overexpression promotes metabolic adaptation under platinum stress, conferring resistance both in vitro and in vivo [101].

b) Lipid Metabolism

Lipid metabolism reprogramming also contributes to chemoresistance in ovarian cancer [102]. Dysregulation of enzymes involved in fatty acid synthesis, polyunsaturated fatty acid (PUFA) metabolism, and phospholipid remodeling has been observed in platinum-resistant tumors [103–105]. For example, overexpression of fatty acid desaturases, or altered cholesterol biosynthesis, may promote membrane remodeling and redox buffering, facilitating survival under drug exposure. Intriguingly, recent studies report a metabolic shift from glycolysis to fatty acid uptake and β-oxidation in cisplatin-resistant cells. This transition supports mitochondrial energy production and limits glycolysis-induced oxidative stress, thereby enhancing survival under chemotherapy [106].

c) Amino Acid Metabolism

Resistance is also shaped by amino acid metabolism, particularly involving GSH and cysteine, which can directly conjugate with platinum compounds to promote drug efflux. Elevated GSH levels in tumor cells and fibroblasts have been linked to decreased intracellular platinum accumulation and enhanced resistance [107]. However, some studies report the opposite—GSH depletion sensitizes cells by increasing intracellular platinum levels and DNA damage [108]. These conflicting results underscore the complexity and context-dependence of metabolic resistance mechanisms, and further investigation is warranted to clarify the therapeutic implications.

Together, these metabolic alterations highlight how platinum-resistant ovarian cancer cells exploit bioenergetic and redox pathways to support treatment evasion. Targeting metabolic vulnerabilities is therefore emerging as a promising strategy to restore platinum sensitivity.

#### Epigenetic modulation

Epigenetic plasticity plays a pivotal role in the emergence of drug-tolerant persister (DTP) cells—a quiescent, reversible state that allows tumor cells to survive platinum chemotherapy and drive relapse [76, 109–113]. Epigenetic modifications do not alter the DNA sequence but regulate gene expression programs associated with survival, stemness, and repair.

- DNA Methylation and Histone Modification.

Two major epigenetic mechanisms contribute to platinum resistance: DNA methylation, particularly at promoter CpG islands, can silence tumor suppressor genes or activate drug resistance pathways. Histone modifications, such as acetylation and methylation, remodel chromatin accessibility and transcriptional responses to therapy. Recent studies have identified several genes modulated by these epigenetic changes in platinum-resistant ovarian cancer. *RIPK4*, *IFFO1*, *PTGER2*, *and BIRC5* have been linked to methylation changes that promote resistance and stemness features [114–117]. Liquid biopsy techniques now allow detection of epigenetic changes in circulating tumor DNA (ctDNA). For instance, hypermethylation of *NKAPL* in plasma samples has been associated with poor platinum response, offering a potential biomarker for noninvasive monitoring [32].

b) Epigenetic Regulators

Research has also focused on epigenetic regulators. For example, the H3K79 methyltransferase DOT1L has been identified as a key regulator of β-catenin in ovarian cancer stem cells, contributing to drug resistance [112]. hMOF, a MYST family histone acetyltransferase involved in post-translational chromatin modification, has been shown to confer cisplatin resistance in multiple ovarian cancer cell lines and xenograft models by promoting p53 degradation [118].

These findings suggest that platinum resistance is not solely a genetic phenomenon but also a consequence of epigenetically controlled transcriptional plasticity. Targeting histone modifiers and DNA methylation pathways—alone or in combination with platinum—represents a promising therapeutic strategy to eliminate residual tumor cells and prevent relapse.

### Tumor microenvironment contributions

**Fig. 2:**
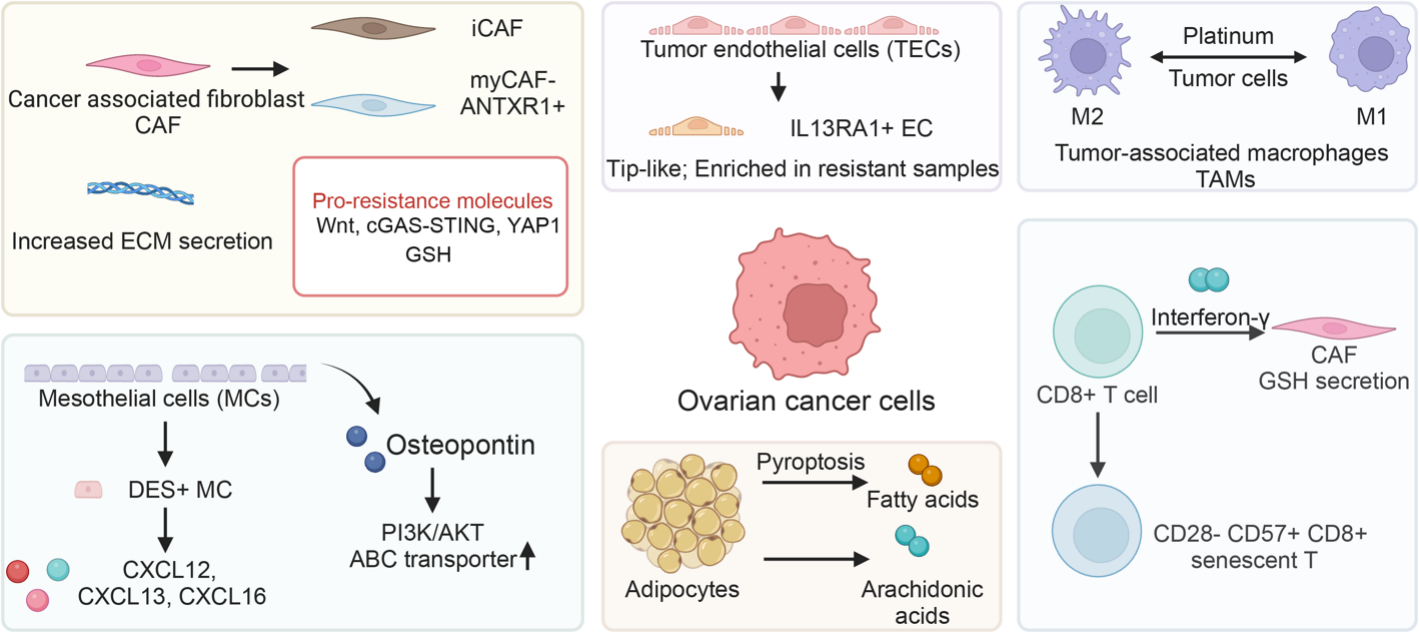
Tumor Microenvironment and Platinum Resistance in Ovarian Cancer. Graphical overview of the tumor microenvironment (TME) influencing platinum resistance in ovarian cancer. The stroma, predominantly composed of cancer-associated fibroblasts (CAFs), contributes to resistance through extracellular matrix (ECM) secretion, increased tumor stiffness, and cytokine release, involving pathways like FAK, β1 integrin-pMLC-YAP, Wnt, cGAS-STING, and YAP1. Subtypes of CAFs, such as CAF-S1 and inflammatory CAFs (iCAFs), play distinct roles in resistance mechanisms. Mesothelial cells (MCs), classified into subtypes like DES+ MCs, secrete soluble factors like osteopontin, interacting with cancer cells to enhance chemoresistance. Tumor endothelial cells (TECs) create an immunosuppressive niche, with tip TECs associated with platinum resistance. Adipocytes in omental tissues release free fatty acids, such as arachidonic acid, promoting resistance through the Akt pathway. Immune cells, including CD8+ T cells and tumor-associated macrophages (TAMs), exhibit complex interactions within the TME. CD8+ T cells release interferon-γ, affecting fibroblast-mediated resistance, while TAMs show plasticity between M1 and M2 subtypes, influenced by chemotherapy. [created with BioRender.com (https://biorender.com/)]

While tumor-intrinsic mechanisms are central to platinum resistance, mounting evidence underscores the pivotal role of the TME in modulating therapeutic response. The TME not only provides structural support but also functions as a biochemical and immunological niche that nurtures residual disease and fosters drug tolerance. Clinically, a tumor-stroma proportion (TSP) > 50% has been associated with poorer outcomes and higher likelihood of chemoresistance in ovarian cancer [119]. Spatial heterogeneity within the TME—particularly among distinct subclonal regions—further complicates therapeutic targeting, as individual tumor clones may reside in differentially permissive microenvironments [120]. Moreover, malignant ascites, rich in soluble factors and stromal cells, have been shown to promote chemoresistance and metastatic dissemination [121]. Deciphering how the TME protects residual tumor cells is therefore critical to understanding platinum resistance and developing combinatorial treatment strategies (Fig. 2).

#### Stroma compartment

- Cancer-Associated Fibroblasts (CAFs)

CAFs are one of the most abundant stromal cell types in ovarian cancer and are key facilitators of platinum resistance. Through both mechanical remodeling and paracrine signaling, CAFs create a tumor-favoring microenvironment that limits drug efficacy and promotes survival.

One major mechanism by which CAFs influence resistance is via extracellular matrix (ECM) remodeling. CAFs deposit ECM proteins that increase tissue stiffness and interstitial pressure, thereby hindering platinum drug penetration into the tumor core [122, 123]. Biophysical studies using polyacrylamide hydrogels and shear wave elastography have confirmed that ECM stiffness positively correlates with chemoresistance in ovarian cancer [124, 125]. Mechanistically, ECM stiffness activates integrin signaling pathways. A study by Pietilä et al. demonstrated that specific ECM cues engage the β1 integrin–FAK–pMLC–YAP axis, which enhances survival signaling and promotes platinum tolerance [126]. This pathway integrates mechanotransduction with transcriptional plasticity, enabling cancer cells to resist apoptosis in a stiffened microenvironment.

In addition to mechanical effects, CAFs secrete pro-survival cytokines and chemokines that reinforce platinum resistance. These include: Wnt ligands, which enhance CSC features and promote stemness-associated resistance; cGAS-STING pathway activators, contributing to immunosuppression and inflammation-induced survival; YAP1 inducers, which promote transcriptional reprogramming in cancer cells toward a drug-tolerant phenotype [127–129]. Beyond cytokines and chemokines, CAFs also contribute metabolic support. They secrete GSH and cysteine, which are taken up by tumor cells to detoxify platinum compounds—especially in the presence of CD8 + T cells that would otherwise suppress these effects [107].

Recent advances in scRNA-seq have revealed the heterogeneity of CAFs, enabling classification into functionally distinct subtypes—CAF-S1 to CAF-S4 [95]. Among these, CAF-S1 (*ANTXR1*+) subtypes are particularly enriched in platinum-resistant tumors. They not only promote drug tolerance but also suppress CD8 + T cell cytotoxicity, exacerbating immune evasion and resistance [129]. Other subsets, such as inflammatory CAFs (iCAFs)—marked by high IL6 and CXCL12 expression—have also been implicated in maintaining drug-tolerant cancer cell states [21]. Interestingly, CAF-S1 (*ANTXR1*^+^) cells often express FAP and resemble myofibroblastic CAFs (myCAFs), raising intriguing questions about the functional overlap and division of labor between iCAFs and myCAFs in modulating platinum response [129]. Understanding the CAF subtype–specific contributions to chemoresistance remains a major research frontier. Disrupting their mechanical or biochemical interactions with tumor cells holds potential for sensitizing ovarian cancers to platinum therapy and breaking immune suppression.

b)  Mesothelial cells (MCs)

Mesothelial cells (MCs), which line the peritoneal cavity and omental surfaces, have emerged as active contributors to the development of platinum resistance in ovarian cancer. Far from being inert barriers, MCs secrete a variety of soluble factors that influence tumor cell behavior and drug response. One prominent example is osteopontin, a glycoprotein abundantly secreted by MCs. Osteopontin has been shown to promote platinum resistance by enhancing stemness, activating the PI3K/AKT signaling pathway, and upregulating ABC transporter expression, thereby increasing drug efflux and reducing intracellular platinum accumulation [130].

Additionally, bidirectional signaling between tumor cells and MCs appears to modulate resistance. For instance, JAG2-overexpressing ovarian cancer cells can activate Notch receptors on MCs, leading to increased IL-6 secretion, which in turn promotes tumor cell survival and chemoresistance [79]. Recent scRNA-seq studies have further dissected MC heterogeneity, identifying at least four transcriptionally distinct subtypes: *DES*^+^ MCs, *VCAN*^+^ MCs, *CXCL10*^+^ MCs, and *MKI67*^+^ MCs [131]. Among these, *DES*^+^ MCs are enriched in ascites and exhibit high secretory activity, producing chemokines such as CXCL12, CXCL13, and CXCL16. These molecules potentially foster tumor-stroma crosstalk and promote a chemoresistant niche [131].

Despite these insights, the specific roles of MC subtypes in mediating platinum resistance remain poorly defined. Future studies should focus on elucidating the functional relevance of each MC population, particularly in the context of ascitic microenvironments and tumor-immune interactions.

c) Endothelial cells

Tumor endothelial cells (TECs) constitute another important but underexplored component of the ovarian cancer microenvironment. Unlike normal vasculature, TECs in tumors are structurally abnormal, functionally immature, and frequently contribute to immunosuppression and nutrient redistribution, all of which can foster therapeutic resistance [132, 133].

Recent single-cell profiling has identified multiple TEC phenotypes, including “tip-like” TECs, which are characterized by high migratory and angiogenic activity and correlate with poor clinical outcomes across various cancer types [134, 135]. In ovarian cancer specifically, a tip-like TEC subset expressing *IL13RA1* was found to be enriched in platinum-resistant tumors, suggesting a potential role in resistance through niche remodeling and immune modulation [131].

However, mechanistic data on TEC-driven platinum resistance in ovarian cancer remain scarce. Further functional validation is necessary to determine whether TECs actively confer drug resistance or merely reflect aggressive tumor phenotypes. Exploring this axis could open new therapeutic opportunities targeting tumor vasculature.

d) Adipocytes

Adipocytes, particularly those in the omentum—a primary metastatic site for ovarian cancer—play a significant role in tumor progression and drug resistance. It is estimated that up to 80% of advanced ovarian cancer cases involve omental metastasis [136, 137]. One mechanism by which adipocytes promote resistance is through pyroptosis-induced lipid release, wherein dying adipocytes liberate free fatty acids that are then taken up by adjacent tumor cells and provide a metabolic substrate that supports survival and reduces platinum sensitivity [138].

Lipidomic profiling has further identified arachidonic acid as a chemo-protective lipid. Arachidonic acid directly stimulates ovarian cancer cells, activating the PI3K/AKT pathway and enhancing resistance to platinum-based chemotherapy [139].

These findings highlight the metabolic coupling between tumor cells and adipocytes in the peritoneal cavity. Disrupting fatty acid transfer or signaling may thus represent a novel approach to overcoming resistance in adipocyte-rich metastatic environments.

#### Immune compartment

The role of the immune system in chemoresistance within ovarian cancer is intricate and multifaceted. Immune cells, including both lymphoid and myeloid populations, can either support tumor immunity or contribute to resistance by creating an immunosuppressive niche. The balance between immune surveillance and immune evasion plays a crucial role in the development and progression of platinum resistance. While some immune components can enhance chemoresistance, others may counteract it, depending on the context and the immune regulation within the TME. This section reviews the critical immune cell populations that influence platinum resistance, focusing on T cells and macrophages, and their complex interplay within the ovarian cancer ecosystem.

- T cells

Recent studies have highlighted distinct immunological profiles between resistant and sensitive ovarian cancer patients, revealing an immune-inflamed phenotype in tumors after chemotherapy [140–142]. Among the most studied immune infiltrates, CD8^+^ T cells play a significant role in platinum response. Their presence correlates with prolonged survival in patients undergoing cisplatin treatment, as they help maintain anti-tumor immunity by secreting IFN-γ and directly killing cancer cells [143].

Interestingly, CD8^+^ T cells also influence the TME by altering the metabolic landscape of surrounding stromal cells. For example, IFN-γ released by activated CD8 + T cells can counteract fibroblast-mediated chemoresistance by targeting GSH and cysteine metabolism, which are crucial for maintaining platinum resistance in fibroblasts [107].

However, immune evasion mechanisms in ovarian cancer also complicate this interaction. Plasma gelsolin, secreted by tumor cells, has been shown to induce apoptosis in CD8 + T cells, thereby suppressing their ability to produce IFN-γ and fostering a pro-survival environment within the tumor [144]. Additionally, a subset of senescent CD8^+^ T cells, marked by CD28^−^ CD57^+^, has been linked to long-term survival and therapy response in high-grade serous ovarian cancer patients, particularly in ascitic fluid. These senescent T cells may represent a novel target for enhancing immune responses and overcoming platinum resistance [145].

A notable consideration is that immunological profiles may vary between primary and metastatic sites within the same patient. The high mutational burden and transcriptional diversity of ovarian cancer cells can lead to different immune microenvironments, even within a single tumor [120, 146, 147]. This adds another layer of complexity to the immune-tumor interaction in platinum resistance.

b) Macrophages

Tumor-associated macrophages (TAMs) represent a dominant component of the myeloid compartment in the ovarian cancer TME and play a pivotal role in chemoresistance. These cells exhibit remarkable plasticity, with M1 TAMs generally having anti-tumor properties and M2 TAMs promoting immune suppression, tissue remodeling, and tumor progression [148].

Chemotherapy itself can influence TAM polarization, with studies showing that ovarian cancer cells promote the M2 phenotype under treatment conditions [149]. Interestingly, both resistant and sensitive ovarian cancer cells can drive this M2 polarization, suggesting that factors beyond chemotherapy itself, such as tumor-derived signals, influence this process [150]. However, some studies indicate that chemotherapy can also reprogram TAMs back to an anti-tumor phenotype, highlighting the dynamic nature of TAM behavior. Research using orthotopic mouse models has shown that chemotherapy can induce reprogramming of TAMs toward a more pro-inflammatory, anti-tumor profile, which may enhance therapeutic efficacy and suppress chemoresistance [151].

This apparent contradiction in TAM behavior underscores the complexity of their role in the TME. It is likely that multiple TAM phenotypes coexist within ovarian cancer tumors, with the specific impact on resistance largely dependent on the niche in which the macrophages reside. The factors that regulate TAM polarization in the TME—such as hypoxia, nutrient availability, and cytokine signaling—are areas of active research and represent potential therapeutic targets for overcoming resistance.

## State-of-art methodologies for studying platinum resistance

**Fig. 3:**
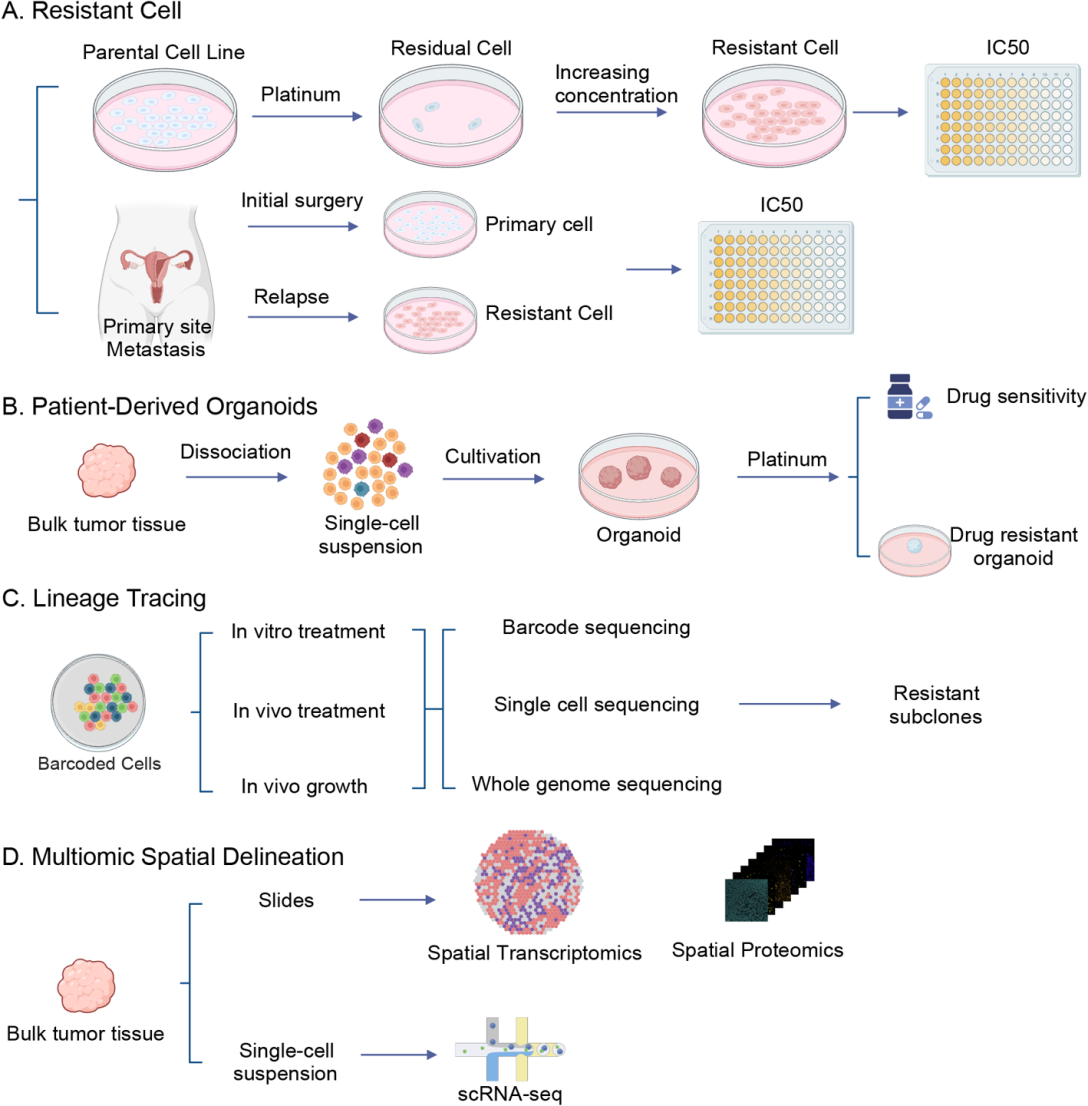
Methodologies for Studying Platinum Resistance in Ovarian Cancer. **A**. Generation of platinum-resistant cells: Resistant cells can be derived from established cell lines through gradual exposure to increasing platinum concentrations, or from paired primary patient samples obtained before and after the development of platinum resistance. **B**. Patient-derived organoids: Ovarian cancer organoids, developed from patient tissue, serve as valuable models for drug sensitivity testing and can be used to generate platinum-resistant organoids, mimicking in vivo resistance development. **C**. Lineage tracing: Advanced lineage tracing techniques enable the identification and tracking of evolving or pre-existing resistant cell populations, providing insights into the dynamics of resistance development. **D**. Multi-omics spatial analysis: Cutting-edge spatial multi-omics, including spatial transcriptomics, spatial proteomics and scRNA-seq, allow for the delineation of spatial contexts that contribute to the formation and maintenance of platinum-resistant cells. These methods provide a comprehensive view of the tumor microenvironment and its role in chemoresistance. [created with BioRender.com (https://biorender.com/)]

Studying platinum resistance in ovarian cancer presents significant methodological challenges due to the complexity and heterogeneity of the phenomenon (Fig. 3).

### Resistant cell lines

While chemoresistant cell lines have long been the cornerstone of in vitro research, variability in the criteria used to define resistance and sensitivity has highlighted the need for standardization in the field [94]. Utilizing patient-derived cell lines, such as PEO1 and PEO4, which exhibit different responses to platinum therapy, provides a more clinically relevant model to study resistance mechanisms. Furthermore, tumor spheroids or cancer stem cells have been valuable for investigating resistance linked to stemness. However, these models may not fully recapitulate the clinical progression of resistance, particularly as it evolves through multiple lines of chemotherapy.

### Patient-derived organoids (PDOs)

PDOs have emerged as a promising tool for studying chemoresistance and predicting drug responses in ovarian cancer. These organoids retain key tumor features, including heterogeneity and stem cell-like properties, making them a more accurate representation of the original tumor compared to traditional 2D cell cultures. PDOs have been instrumental in understanding the genetic and epigenetic landscape of resistance, providing personalized models for testing drug combinations and identifying patient-specific resistance patterns [152, 153].

Despite these advantages, PDOs are not without limitations. They consist mainly of tumor cells and lack the full complexity of the TME, including non-tumor cell populations such as fibroblasts, immune cells, and endothelial cells, which can influence resistance mechanisms. scRNA-seq has demonstrated that while PDOs mimic many aspects of the original tumor, they do not replicate the full TME interaction [154]. Therefore, while PDOs serve as an invaluable tool for understanding the intrinsic properties of chemoresistant tumor cells, they cannot fully capture the extrinsic factors that contribute to resistance. Continued efforts to incorporate TME components into organoid models, such as co-culturing with stromal or immune cells, will enhance their utility in resistance studies.

### Lineage tracing and clonal evolution

Recent advancements in lineage tracing technologies have provided groundbreaking insights into the evolution of chemoresistant cells. Techniques such as ReSisTrace [12], macsGESTALT [155], Rainbow [156, 157], DNA barcodes [158] and Watermelon [22] allow for tracking the origin and clonal evolution of resistant or metastatic cells with unprecedented precision [159]. These tools enable researchers to identify pre-existing resistant clones and follow their dynamics during treatment, revealing how specific populations evolve in response to chemotherapy.

Additionally, in vivo barcoding technologies, including those using adeno-associated viruses (AAVs), enable the tracking of tumor evolution in more physiologically relevant contexts [160]. By tagging individual cells or small populations, these technologies provide a comprehensive view of clonal survival and genetic drift during treatment, offering insights into the heterogeneity of resistance and how it develops over time.

These technologies, when combined with high-throughput sequencing and drug perturbation assays, have the potential to uncover novel resistance pathways and help identify predictive biomarkers for resistance. However, further optimization and validation in clinical models are needed to translate these findings into actionable insights.

### Single cell RNA sequencing (scRNA-seq)

Advances in scRNA-seq have greatly deepened our understanding of intra-tumor heterogeneity in ovarian cancer. Applications of scRNA-seq in ovarian cancer cohorts have uncovered subpopulations of tumor, stromal, and immune cells with distinct gene expression programs linked to prognosis or therapeutic response [161]. For instance, longitudinal scRNA-seq studies in high-grade serous ovarian carcinoma revealed that residual tumor cells adopt stress-response transcriptional states that may pre-dispose them to later resistance [21]. Another group demonstrated that chemo-induced overexpression of stabilin-1 in macrophages and FOXP3 in Tregs was linked to poor chemotherapy response [162].

### Spatial multi-omics: spatial transcriptomics and spatial proteomics

However, transcriptomic data alone lacks spatial context and does not always reflect protein-level regulation. Recent advances have made it possible to explore tumor microenvironments at both the RNA and protein levels in their original spatial context. Spatial transcriptomics enables mapping of gene expression across tissue sections, revealing how resistant cell subpopulations localize relative to hypoxic zones, stromal compartments, or immune deserts. Spatial transcriptomics has revealed that chemoresistant cells often localize to areas with hypoxia, altered extracellular matrix, and immune suppression, which foster a protective microenvironment [120, 163].

To complement transcript-level data, spatial proteomics techniques like imaging mass cytometry (IMC), cyclic immunofluorescence (cycIF), co-detection by indexing (CODEX), iterative bleaching extends multiplexity (IBEX), and multiplexed ion beam imaging (MIBI) allow simultaneous mapping of dozens of protein markers across individual cells in situ, which reveals how immune and stromal cell phenotypes and cell–cell interactions vary spatially [164]. One particular example is the recently developed Deep Visual Proteomics (DVP) that enables precise profiling of spatial proteomics in ovarian cancer [165].

Combining spatial transcriptomics with spatial proteomics allows a more comprehensive multi-omic view of niches that may drive or sustain platinum resistance. Recent studies in ovarian have already begun applying spatial proteo-transcriptomic profiling to capture this multidimensional landscape [165].

### Moving forward: integrating methodologies

To gain a comprehensive understanding of platinum resistance, it is essential to integrate these cutting-edge methodologies. Future research should prioritize:

- Standardizing resistance definitions across models to allow for more reproducible studies.
- Developing more representative models that incorporate both tumor heterogeneity and microenvironmental interactions, which are critical for recapitulating the full spectrum of resistance mechanisms.
- Leveraging lineage tracing technologies and spatial multi-omics to track clonal evolution and identify critical microenvironmental niches that drive resistance.

By combining these innovative approaches, researchers will be better equipped to unravel the intricate mechanisms of platinum resistance in ovarian cancer, ultimately leading to more effective therapeutic strategies.

## Strategies to overcome platinum resistance

Overcoming platinum resistance in ovarian cancer remains a central challenge in clinical oncology. A growing body of research has focused on strategies that either target tumor-intrinsic resistance mechanisms or reprogram the tumor microenvironment to resensitize cancer cells. Recent advances in DNA repair inhibition, antibody-drug conjugates (ADCs), anti-angiogenic therapy, immunotherapy and targeted drug delivery have yielded promising results in both preclinical and clinical settings. This section summarizes current and emerging approaches being explored to overcome platinum resistance.

### Targeting DNA repair pathways

Since platinum agents exert cytotoxicity via DNA crosslinking, inhibitors of DNA repair—particularly HR and checkpoint signaling—have garnered attention. PARP inhibitors have shown clinical benefit, especially in BRCA-mutant or HR-deficient tumors [166]. However, PARP resistance often coexists with platinum resistance, necessitating combination strategies. ATR, WEE1, and CHK1 inhibitors have been proposed as alternatives to disrupt DNA damage signaling and induce synthetic lethality in platinum-refractory tumors [167, 168].

### Antibody–drug conjugates (ADCs)

ADCs have emerged as a novel class of therapies delivering cytotoxic agents directly to tumor cells via surface antigens [169]. MIRV, targeting folate receptor-α, has demonstrated clinical efficacy in platinum-resistant ovarian cancer. One such ADC that has entered into clinical investigation on platinum-resistant ovarian cancer is MIRV, which targets folate receptor-α. In the single-arm phase II SORAYA study, the objective response rate (ORR) was 32.4% with a median duration of response around 6.9 months [170]. Building on this trial, the global phase III MIRASOL trial randomized patients with FRα-positive, platinum-resistant disease to MIRV versus investigator’s choice chemotherapy—showing significantly improved PFS, OS, and objective response rate with the ADC [14]. Additional ADCs targeting MUC16, NaPi2b, and HER2 are under active investigation [171]. These agents bypass common resistance mechanisms by improving targeted intracellular drug delivery.

### Anti-angiogenic targeted therapy

Anti-angiogenic therapy has long been a cornerstone of ovarian cancer treatment. The pioneering agent bevacizumab was first shown in phase III trials to significantly improve PFS when added to chemotherapy in advanced ovarian cancer [172, 173]. Over time, anti-angiogenic therapy has been extended into the platinum-resistant setting based on results from the phase III AURELIA trial [174]. More recently, in the randomized phase II APPROVE trial, adding apatinib—an oral VEGFR-2 tyrosine kinase inhibitor that offers an alternative to VEGF-antibody based inhibition—to pegylated liposomal doxorubicin (PLD) improved median PFS compared with PLD alone in platinum-resistant recurrent ovarian cancer [175].

### Immunotherapy-based strategies

While immune checkpoint inhibitors have had limited success as monotherapy in ovarian cancer, combination strategies are under development. These include checkpoint blockade plus anti-angiogenic therapy [176], macrophage reprogramming agents such as CD47 inhibitors [177, 178], TME-targeted cytokine inhibitors such as anti-IL6 or anti-TGF-β antibodies [179, 180], and oncolytic virotherapy [181]. These therapies aim to convert the immunosuppressive TME into an immune-responsive state, thereby improving chemotherapy sensitivity. Several of these agents are being evaluated in clinical trials. For instance, the anti-CD47 antibody magrolimab has been tested in ovarian cancer patients in phase I trial yet no robust phase II/III data are presented [177, 178]. Likewise, the nonrandomized VIRO-15 phase II trial evaluated oncolytic immunotherapy priming followed by platinum-doublet ± bevacizumab in heavily pretreated platinum-resistant/refractory ovarian cancer. It reported an ORR of ~ 54% and median PFS of 11.0 months, outperforming historical expectations for this population [181].

### Nanomedicine and drug delivery innovations

Recent advancements in nanomedicine have led to innovative strategies to overcome platinum resistance in ovarian cancer by enhancing drug delivery, increasing bioavailability, and bypassing common resistance mechanisms. One study developed PLGA nanoparticles that co-deliver carboplatin and the SphK1 inhibitor PF543, improving platinum sensitivity by inhibiting survival pathways in resistant cells [182]. Stimuli-responsive hydrogels loaded with platinum-based drugs can be triggered by ultrasound to release drugs directly at tumor sites, enhancing local drug concentration while minimizing side effects [183].

## Concluding remarks

Platinum resistance in ovarian cancer remains a formidable clinical challenge shaped by a multifactorial interplay between tumor-intrinsic adaptations and tumor microenvironmental influences. This review has outlined the key mechanisms underlying chemoresistance, including cellular adaptations, metabolic reprogramming, epigenetic modulations, and the influential role of the tumor microenvironment.

At the cellular level, resistance evolves not only from genetic mutation but from adaptive programs involving stress responses, epigenetic plasticity, and lineage flexibility. Metabolic shifts toward glycolysis or fatty acid oxidation, as well as altered amino acid metabolism, further contribute to drug tolerance. In parallel, epigenetic regulators help orchestrate survival pathways in drug-tolerant persister cells, marking them as emerging therapeutic targets.

Beyond the cancer cell, the TME plays a decisive role in fostering resistance. CAFs, MCs, ECs, adipocytes, and immune subsets all contribute to a protective and immunosuppressive niche that shields tumor cells from platinum-induced damage. The crosstalk between tumor cells and their stromal context is increasingly recognized as both a barrier and a therapeutic opportunity.

To effectively overcome platinum resistance, future efforts must move beyond single-target approaches and embrace combinatorial strategies that address both cell-intrinsic vulnerabilities and extrinsic microenvironmental support systems. Targeted therapies, immunotherapies, metabolic modulators, and nanomedicine platforms all hold promise, especially when deployed within integrated treatment regimens.

Furthermore, the application of advanced tools such as single-cell multi-omics, spatial transcriptomics, lineage tracing, and organoid models will be crucial in unraveling resistance dynamics and guiding precision medicine. Liquid biopsy-based monitoring may soon enable real-time assessment of resistance evolution, allowing for adaptive therapeutic strategies tailored to the evolving tumor landscape.

In summary, platinum resistance in ovarian cancer is not a fixed trait but a dynamic, evolving process driven by tumor heterogeneity and microenvironmental adaptation. Progress will depend on our ability to integrate biological insight with clinical innovation. With continued investment in translational research, the goal of personalized, resistance-proof treatment strategies is within reach—bringing new hope to patients with this challenging disease.

## Data Availability

No datasets were generated or analysed during the current study.

## Abbreviations

ABC: ATP-binding cassette
ADM: Adrenomedullin
ADC: Antibody–drug conjugate
APC: Antigen presenting cell
CAF: Cancer-associated fibroblast
CBP: Carboplatin
CSC: Cancer stem cell
DDR: DNA damage response
DFS: Disease-free survival
DTP: Drug-tolerant persister
ECM: Extracellular matrix
EMT: Epithelial–mesenchymal transition
EOC: Epithelial ovarian cancer
FTE: Fallopian tube epithelium
GSH: Glutathione
GST: Glutathione-S-transferase
HGSOC: High-grade serous ovarian cancer
HRR: Homologous recombination repair
IMC: Imaging mass cytometry
MC: Mesothelial cell
MMR: Mismatch repair
MT: Metallothionein
NER: Nucleotide excision repair
OXPHOS: Oxidative phosphorylation
PARP: Poly(ADP-ribose) polymerase
PDO: Patient-derived organoid
PFS: Progression-free survival
PUFA: Polyunsaturated fatty acid
scRNA-seq: Single-cell RNA sequencing
TAM: Tumor-associated macrophage
TEC: Tumor endothelial cell
TME: Tumor microenvironment
TSP: Tumor–stroma proportion
